# Virtual Node-Driven Cloud–Edge Collaborative Resource Scheduling for Surveillance with Visual Sensors

**DOI:** 10.3390/s25020535

**Published:** 2025-01-17

**Authors:** Xinyang Gu, Zhansheng Duan, Guangyuan Ye, Zhenjun Chang

**Affiliations:** 1 Center for Information Engineering Science Research, Xi’an Jiaotong University, Xi’an 710049, China; guxinyang@stu.xjtu.edu.cn (X.G.); 311555@stu.xjtu.edu.cn (G.Y.); 2 Intelligent Control Laboratory, Xi’an Research Institute of High Technology, Xi’an 710025, China; changzj2105@163.com

**Keywords:** virtual node, edge computing, cloud computing, resource scheduling

## Abstract

For public security purposes, distributed surveillance systems are widely deployed in key areas. These systems comprise visual sensors, edge computing boxes, and cloud servers. Resource scheduling algorithms are critical to ensure such systems’ robustness and efficiency. They balance workloads and need to meet real-time monitoring and emergency response requirements. Existing works have primarily focused on optimizing Quality of Service (QoS), latency, and energy consumption in edge computing under resource constraints. However, the issue of task congestion due to insufficient physical resources has been rarely investigated. In this paper, we tackle the challenges posed by large workloads and limited resources in the context of surveillance with visual sensors. First, we introduce the concept of virtual nodes for managing resource shortages, referred to as virtual node-driven resource scheduling. Then, we propose a convex-objective integer linear programming (ILP) model based on this concept and demonstrate its efficiency. Additionally, we propose three alternative virtual node-driven scheduling algorithms, the extension of a random algorithm, a genetic algorithm, and a heuristic algorithm, respectively. These algorithms serve as benchmarks for comparison with the proposed ILP model. Experimental results show that all the scheduling algorithms can effectively address the challenge of offloading multiple priority tasks under resource constraints. Furthermore, the ILP model shows the best scheduling performance among them.

## 1. Introduction

Cloud computing and the Internet of Things (IoT) have revolutionized digital systems, endowing them with advanced perception and computational capabilities [1,2]. This transformative synergy has driven the development of innovations such as smart cities [3,4,5], smart grids [6], smart buildings [7], and smart transportation [8], fundamentally reshaping production processes and daily life.

The advancement of IoT technology has significantly accelerated the development of video surveillance systems, enhancing their performance [9]. The number of cameras integrated into IoT systems is rapidly increasing and is projected to reach an astounding 13 billion by 2030 [10], while traditional IoT data, such as humidity, pressure, or temperature, can be seamlessly processed through offloading tasks to the cloud center, the handling of visual sensor data introduces far more complex challenges. Unlike conventional IoT data, visual data are characterized by their high volume, real-time requirements, and the demand for huge computational resources [11].

Cloud computing requires data from IoT devices to be transferred to cloud data centers for processing. This reliance on centralized infrastructure can lead to potential network congestion and delays due to routing issues, resulting in slow response times [12,13]. The frequent transmission of large data sets, especially in applications like video surveillance, consumes large amounts of bandwidth, making it both costly and inefficient. Edge computing, positioned closer to user nodes, significantly reduces transmission latency, conserves network resources, and minimizes data traffic across the network [14,15,16]. By leveraging these latency advantages, edge computing enhances both the QoS and the quality of experience (QoE) for users.

The three-tier edge computing architecture, consisting of the user layer, edge layer, and cloud layer, introduces far more complexity compared to the traditional two-tier cloud-end architecture. This complexity poses substantial challenges for resource scheduling, as it must account for the unique characteristics and interactions of each layer. Efficient resource management in edge computing requires addressing multiple factors, including minimizing delays, managing limited resources, ensuring data security, adapting to geographical constraints, balancing workloads, conserving energy, and maintaining high QoS [17]. To address these challenges, existing resource scheduling algorithms have been extensively studied and have demonstrated promising results in reducing energy consumption in edge devices [18,19,20,21,22], lowering latency for real-time applications [23,24,25,26,27,28,29], and enhancing QoS [30,31,32] for reliable and efficient service delivery.

However, despite significant advancements in optimizing energy, time delay, and QoS, a critical challenge that remains under-explored is the inherent resource scarcity at edge nodes. Edge devices and private clouds typically have limited computational power, storage capacity, and energy resources compared to centralized public cloud data centers. These constraints necessitate intelligent resource management strategies to meet the growing demands of modern applications.

In addition to resource scarcity, another important factor that has received relatively less attention is task prioritization, which refers to the process of assigning different priorities to tasks based on their importance or urgency. In security-related video surveillance systems, different tasks may have varying levels of importance and urgency. Properly prioritizing tasks is crucial to ensure that critical applications, such as real-time threat detection and alert generation, receive the necessary resources and processing power without undue delay.

Our work significantly differs from and improves upon existing studies on three key aspects. First, our algorithm addresses locally non-executable problems, which are particularly common in the field of video surveillance. However, the existing algorithms primarily address locally executable problems, where computing requirements can be solved by users. In contrast, the locally non-executable problem exacerbates the issue of resource scarcity, a challenge that has received limited attention in previous work. Second, the existing algorithms pay little attention to inherent priority differences among tasks, but our algorithm can address varying task priorities in video surveillance. Third, our algorithms use user–edge–cloud three-layer computing architecture, which is more general than user–edge or user–cloud architectures. For instance, Deng et al. [23] noted packet losses but overlooked task priority using only a user–edge architecture. Lyu et al. [20] considered task priority but ignored resource scarcity using only a user–edge architecture. Li et al. [31] addressed both resource scarcity and task priority, but their user–edge architecture did not account for the memory and GPU constraints.

We aim to address two critical issues in clould–edge collaborative resource scheduling, the shortage of computing resources at edge nodes and the prioritization of tasks, within the edge computing framework in this paper. We propose a novel resource scheduling approach that dynamically allocates resources considering both the priority and resource requirements of tasks, thereby optimizing the overall performance of the edge computing platform. By focusing on both aspects, our work contributes to ongoing efforts to enhance the capabilities and reliability of edge computing systems, paving the way for more robust and responsive applications. We refer to this challenge as the priority-based Edge User Allocation under Resource Scarcity (EUARS) problem and make the following main contributions in this paper:

(1) We formally define the concept of a virtual node and show its effectiveness in solving priority-based EUARS problems.
(2) We propose a convex-objective virtual node-driven integer linear programming (ILP) model/algorithm and demonstrate its efficiency.
(3) We extend the random algorithm, genetic algorithm, and heuristic algorithm using the virtual node concept. These three virtual node-driven algorithms work as benchmark algorithms to demonstrate the effectiveness, flexibility, and superior performance in terms of success rate and edge resource utilization of the proposed virtual node-driven ILP algorithm.

The rest of this paper is organized as follows. Section 2 reviews the related work. Section 3 defines the EUARS problem and presents the virtual node approach. Section 4 describes the virtual node-driven ILP algorithm. Section 5 describes the virtual node-driven random, genetic, and heuristic algorithms. Section 6 details the experiments and the analysis of their results. Section 7 concludes the paper and discusses future research endeavors.

## 2. Related Work

The main goal of resource scheduling in edge computing is to offload computational tasks to the edge, closer to the user. This can significantly reduce transmission latency and alleviate the amount of data transmitted on a network. This section will focus on resource scheduling targets, with particular emphasis on two key aspects: latency or time delay, and energy consumption. In comparison, other resource scheduling targets, such as QoS, cost, and resource utilization, are considered as complementary improvements to latency and energy efficiency. We have also summarized the key features of related work to facilitate a comparison with our proposed algorithms, as shown in Table 1.

### 2.1. Time Delay

Deng et al. proposed the dynamic parallel computing offloading and energy management (DPCOEM) algorithm based on Lyapunov optimization with the goal of minimizing response time and achieved near-optimal performance [23]. Xu et al. aimed to minimize computational latency under long-term energy consumption constraints and proposed an online algorithm named OREO (Online seRvice caching for mobile Edge cOmputing) by combining Lyapunov optimization with Gibbs sampling [24]. Qiao et al. developed a mathematical model aimed at minimizing a weighted combination of task latency and energy consumption in terminal devices [25]. To solve the model and achieve optimal resource scheduling, they employed a differential immune process integrated with the Deep Deterministic Policy Gradient algorithm. Xing et al. proposed a model to minimize computational latency under energy and frequency constraints, solving the complex Mixed-Integer Nonlinear Programming (MINLP) problem using a variable relaxation approach combined with a greedy joint optimization strategy to reduce complexity [26]. Xu et al. developed a multi-objective optimization model to address computational offloading, focusing on the trade-off between execution time and energy consumption for mobile devices. To solve this optimization problem, they employed the Non-Dominated Sorting Genetic Algorithm III (NSGA-III) [27]. Yu et al. proposed a fine-grained collaborative computation offloading and caching strategy, leveraging caching to enhance offloading decisions and achieve the scheduling objective of minimizing the total execution delay for mobile users within the network [28]. Huang et al. proposed a model to minimize latency in a multi-replica storage architecture [29]. They developed a heuristic method to efficiently solve the model, achieving satisfactory results even in large-scale problem instances.

### 2.2. Energy Consumption

Ning et al. proposed an energy-efficient scheduling framework that addresses the challenges of task scheduling and downlink energy consumption through the application of a heuristic algorithm [18]. Chen et al. introduced an energy-efficient device-to-device (D2D) crowd offloading framework that minimizes the total power consumption of collaborative task execution among devices using a graph-matching-based optimal scheduling policy [19]. Lyu et al. proposed a request-and-admission framework that implements selective offloading strategies to minimize energy consumption in IoT devices while ensuring compliance with diverse service latency requirements [20]. Yang et al. investigated the problem of minimizing energy consumption while ensuring that latency stays within an acceptable range [21]. They formulated the problem as a mixed-integer linear programming (MILP) model and proposed a Benders decomposition-based solution for it. Li et al. aimed to minimize energy consumption by leveraging the Successive Convex Approximation (SCA) method and the Dinkelbach algorithm to transform the non-convex problem into a solvable form, ultimately achieving the most energy-efficient computational offloading strategy for unmanned aerial vehicles (UAVs) [22].

### 2.3. Other Targets

Badri et al. aimed to maximize the total QoS of the system under an energy budget constraint [30]. They formulated the problem as a multi-stage stochastic application placement problem and employed the Sample Average Approximation (SAA) method to address it. Li et al. proposed a QoS quantification scheme based on a statistical computation model and a statistical transmission model [31]. They formulated the QoS constraint problem as an MINLP problem and addressed it using convex optimization theory and Gibbs sampling. Lai et al. abstracted the QoE-aware Edge User Allocation (EUA) problem and proved that the EUA problem is NP-hard [32]. To address this, they proposed an optimization method using ILP and additionally introduced a heuristic approach as an alternative solution.

## 3. Video Surveillance System Architecture

**Figure 1:**
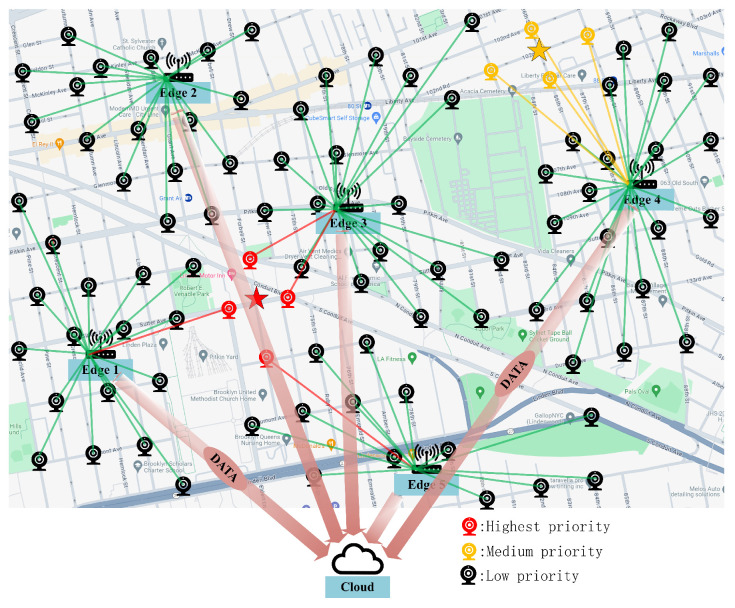
Video surveillance scene diagram. The red star indicates areas where security incidents have occurred, while the yellow star indicates areas with a high likelihood of future security incidents. Red cameras that can monitor red star areas have the highest priority, while yellow cameras that can monitor yellow star areas have the medium priority. The line between cameras and edge servers represents wired network connections.

The architecture of the video surveillance system described in this paper comprises *n* surveillance cameras, *m* edge computing boxes, and a centralized cloud server, as illustrated in Figure 1. The surveillance cameras are connected to the edge computing boxes via wired connections, which are also similarly linked to the cloud server through wired networks. The surveillance cameras are solely responsible for generating video stream monitoring data and do not have any computational or analytical capabilities. Consequently, the computational tasks of the surveillance cameras must be offloaded to either the edge computing boxes or the cloud server.

For such an application, security incidents occur at various locations and are monitored by the surveillance camera closest to the incident site. When a camera detects a security incident, it transitions to emergency mode, which demands significantly more computational resources, including CPU cores, GPU blocks, memory capacity, and bandwidth, to handle the emergency tasks effectively. Security incidents often exhibit certain precursors during the transition from non-occurrence to occurrence. During this intermediate stage, the camera transitions to an alert mode, which consumes fewer computational resources than emergency tasks but more than regular tasks. Introducing the alert mode enhances the accuracy of emergency task detection and reduces the latency in responding to incidents. By optimizing the allocation of emergency, alert, and regular tasks across the system, we ensure that the system can respond to emergencies quickly and with low latency under various conditions, providing excellent computational services for high-priority tasks. A key objective of the EUARS problem is to ensure the efficient and prioritized execution of high-priority tasks.

In the context of this study, video stream monitoring data are transmitted via wired connections, and as such, wireless network latency models are not considered. We simplify latency by categorizing tasks: those executed on edge computing boxes are regarded as low-latency tasks, while those offloaded to the cloud server are regarded as high-latency tasks. A key objective of the EUARS problem is to minimize the total system latency. Unlike typical mobile edge computing (MEC) systems, surveillance cameras do not have independent computational capabilities, setting them apart from mobile devices. A fundamental solution in MEC scenarios often involves completing all computational tasks locally on the mobile device, thereby ensuring the problem remains solvable. However, in our system architecture, large-scale security incidents can lead to insufficient computational or network resources, inevitably resulting in some tasks being left uncompleted.

**Figure 2:**
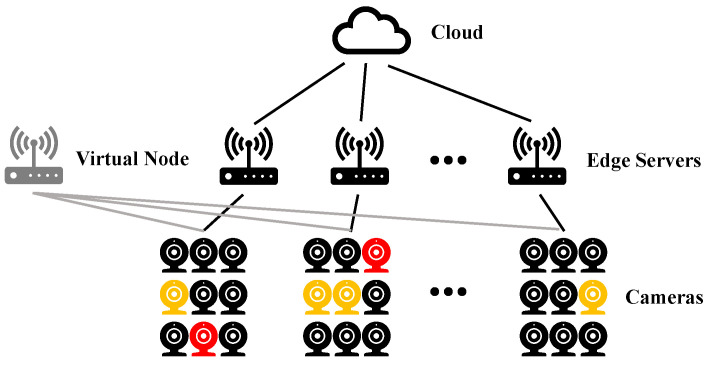
Video surveillance task topology diagram: the gray node is the virtual node. The connection between the camera and edge servers indicates that tasks generated by the camera can be offloaded to the corresponding edge server or further offloaded to the cloud. A virtual node is connected to all cameras, enabling any task generated by the cameras to be offloaded to it. The virtual node is distinct from edge servers and the cloud, serving as a conceptual entity.

To tackle the challenge of optimal task scheduling under network congestion and resource constraints, we introduce the concept of a **virtual node**, as illustrated in Figure 2. The virtual node is connected to all surveillance cameras, allowing tasks from any camera to be seamlessly offloaded to it. The virtual node is modeled as having unlimited computational resources and network bandwidth. As a conceptual entity, it requires no physical wiring between cameras and the virtual node. The virtual node remains physically disconnected from both edge computing boxes and cloud servers. In real-world scenarios, tasks abandoned due to resource constraints correspond conceptually to those offloaded to the virtual node. Although the introduction of the virtual node increases the complexity of the system’s topology, it ensures the optimal scheduling problem remains solvable. For example, a fundamental solution to the new system architecture is to offload all tasks to the virtual node.

## 4. Virtual Node-Driven ILP Model/Algorithm

We propose a novel task-resource scheduling model to address the EUARS problem. This model assigns varying weights to different task scheduling strategies, enabling a more effective and adaptive allocation of resources. Table 2 summarizes the key variables used in the model along with their corresponding descriptions, providing a comprehensive overview of the foundational elements of our model. Here, we suppose that the proposed ILP model is solved using the Gurobi [33] solver, referred to as the ILP algorithm in the subsequent discussion.

The proposed model aims to maximize the completion rate of higher-priority tasks while minimizing the total system latency for these tasks, as shown in the following equation:

(1) $$\begin{array}{c} \max\sum_{i=1}^{n}g^{p_{i}}QoS\left(i\right) \end{array}$$

(2) $$\begin{array}{c} \text{s.t.}\left\{\begin{array}{cc} C1:\sum_{i=1}^{n}x_{i,j}D_{u_{i},k}\leq D_{s_{j},k}, & \forall k\in \{1,2,3,4\}\\ C2:\sum_{i=1}^{n}y_{i}D_{u_{i},k}\leq D_{c,k}, & \forall k\in \{1,2,3,4\}\\ C3:\sum_{j=1}^{m}x_{i,j}+y_{j}+v_{i}=1, & \forall i\in \{1,2,3...n\}\\ C4:x_{i,j}\in \left\{0,1\right\}\\ C5:y_{j}\in \left\{0,1\right\}\\ C6:v_{i}\in \left\{0,1\right\} \end{array}\right. \end{array}$$

The virtual node plays a critical role in the resource scheduling objective function related to QoS, as reflected in Equation (3). Additionally, its significance is evident in C3 and C6 of Equation (2). For tasks within the same priority level, we do not differentiate further between their individual priorities. To balance the above two objectives and be consistent with real-world video surveillance scenarios, inspired by the work in [32], we reformulate the above problem as follows. Specifically, maximizing the scheduling success rate of high-priority tasks and minimizing total transmission delay are transformed into maximizing the overall Quality of Service (QoS) across all tasks. The QoS of a single task is shown in Equation (3).

(3) $$\begin{array}{c} QoS\left(i\right)=\left(\frac{1}{k}y_{i}+\sum_{j=1}^{m}x_{i,j}-v_{i}\right) \end{array}$$

We introduce two hyperparameters: *g*, determining the weight of tasks with different priorities in the total QoS, and *k*, reflecting the impact of varying transmission delays on QoS. These two hyperparameters enable a flexible trade-off between the two optimization objectives, ensuring practical applicability and relevance to real-world conditions.

In the QoS equation (Equation (3)), *k* is a value greater than 1, designed to differentiate the QoS contributions based on the scheduling location of a task. Specifically, when a task is scheduled to the edge computing box, its QoS value is 1. When a task is scheduled to the cloud server, its QoS value is 1/k. When a task is scheduled to a virtual node, its QoS value is −1. We recommend setting *k* to 4, as it provides a reasonable balance between the relative QoS contributions of different scheduling options.

Now, we consider an extreme scenario: when a high-priority task needs to be processed but requires the full computational resources of an edge computing box. At the same time, a group of low-priority tasks whose cumulative QoS weight exceeds *g* could potentially be scheduled to the same edge computing box. Under such circumstances, the current QoS formulation might prioritize handling multiple less critical tasks instead of the high-priority task.

This outcome is unacceptable within the framework of our EUARS problem, as it contradicts the fundamental objective of prioritizing high-priority tasks. To address this issue, we propose a revised form for *g*, as defined in Equation (4), ensuring that the optimization process be consistent with the intended prioritization scheme. For example, if the edge server has a 24-core or 32-core CPU and the users’ tasks require 1, 2, or 4 cores, then the value of *g* is 32.

(4) $$\begin{array}{c} g=\max_{i,j,k}\frac{D_{s_{j},k}}{D_{u_{i},k}},\forall i\in \left\{1,2...n\right\},\forall j\in \left\{1,2...m\right\},\forall k\in \left\{1,2,3,4\right\} \end{array}$$

Equation (2) defines three key constraints: C1 restricts the resource consumption by a task scheduled to an edge computing box, ensuring it does not exceed the available resources of that box; C2 imposes a limit on the resource consumption of tasks scheduled to the cloud server, ensuring that no resource usage exceeds the total resources available on the server; C3 enforces the condition that each task can only be scheduled on a single device, corresponding to the 0-1 offloading problem in our EUARS problem; C4 indicates that the *i*-th task scheduled to the *j*-th edge computing box can only have two states: scheduled or not scheduled, with no intermediate states; C5 specifies that the *i*-th task scheduled to the cloud server can only have two states, scheduled or not scheduled; C6 similarly denotes that the *i*-th task scheduled to the virtual node can only have two states: scheduled or not scheduled.

In constraint C3, without introducing the virtual node, either $x_{i,j}$ or $y_{j}$ must be nonzero. Under conditions of resource scarcity, this requirement can be in conflict with the limitations imposed by constraints C1 and C2, rendering the entire optimization problem infeasible. Consequently, it would be impossible to obtain a feasible solution using a solver. By introducing the virtual node, both $x_{i,j}$ and $y_{j}$ are allowed to be zero simultaneously, indicating that the task is scheduled to the virtual node and C3 is no longer in conflict with the constraints of C1 and C2.

**Figure 3:**
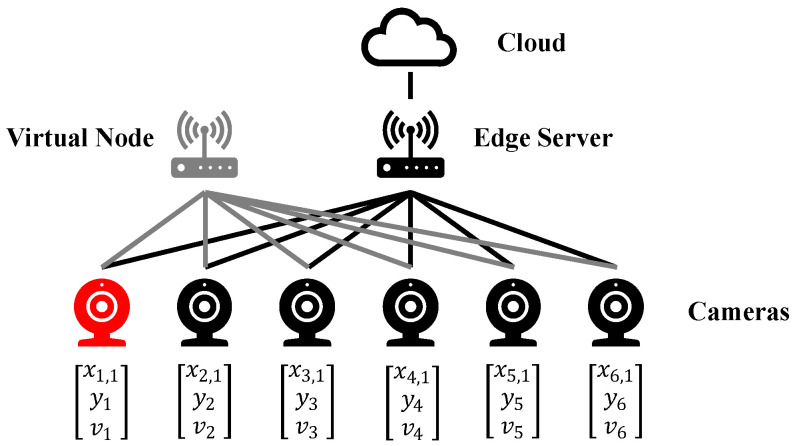
A brief example demonstrating how the ILP model works. The red camera generates a high-priority task, while the other five black cameras generate low-priority tasks.

Figure 3 gives a brief example demonstrating how the ILP model handles the task scheduling problem. The scenario consists of a cloud server, an edge computing box, and six cameras, one of which has a high-priority task to be offloaded. We simplify the differences in resource requirements among tasks with varying priorities, assuming that the edge computing box can handle two tasks and the cloud server can handle three. In this example, g=2 and k=4.

The objective function value called "reward", corresponding to the typical solution, is shown in Table 3. In the above scenario, the edge computing box and the cloud server can handle only five tasks, fewer than the total of six tasks. As shown in Table 3, offloading the high priority task to the edge computing box yields the highest reward of 9.5. Offloading it to the cloud server results in a reward of 5, while offloading it to the virtual node yields a reward of −2.5. The optimal solution obtained from the ILP model matches the real-world optimal solution. Offloading the myredhigh-priority task to the edge computing box achieves the lowest latency while fully utilizing the task processing capacity of both the edge computing box and the cloud server.

The proposed objective function, as formulated in Equation (1), transforms our problem into a convex ILP problem. By utilizing an advanced ILP solver, such as IBM ILOG CPLEX [34] or Gurobi [33], this ILP problem can be solved efficiently, enabling the determination of an optimal solution for our priority-based EUARS problem.

## 5. Virtual Node-Driven Comparative Algorithms

To the best of our knowledge, no existing algorithms can be directly used to solve the EUARS problem without significant changes. To demonstrate the superiority of our proposed ILP algorithm, we propose three popular existing optimization algorithms in this section, the random scheduling algorithm, genetic algorithm, and heuristic algorithm, and use them for solving the EUARS problem. Their performance will be compared and analyzed in Section 6 to justify the performance of the ILP algorithm.

### 5.1. Random Scheduling Algorithm

In this algorithm, surveillance camera tasks are randomly assigned to either a directly connected edge computing box or the cloud server, provided that the selected option has sufficient available resources to accommodate the task. If the random assignment selects an edge computing box lacking sufficient resources but the cloud server has adequate capacity, the strategy automatically reallocates the task to the cloud server. Similarly, if the cloud server lacks resources while the edge computing box has sufficient capacity, the task is reassigned to the edge computing box. In cases where neither the cloud server nor the edge computing box has enough resources, the task is assigned to the virtual node.

### 5.2. VND-GA Algorithm

The genetic algorithm (GA), an optimization method inspired by the principles of natural selection and genetic mechanisms, is widely used for solving multi-objective optimization problems. Its advantages include robust global search capabilities, the ability to escape local optima, and independence from derivative information. For the EUARS problem, we propose a virtual node-driven genetic algorithm (VND-GA), as described in Algorithm 1, inspired by NSGA-III in [27].

**Figure 4:**
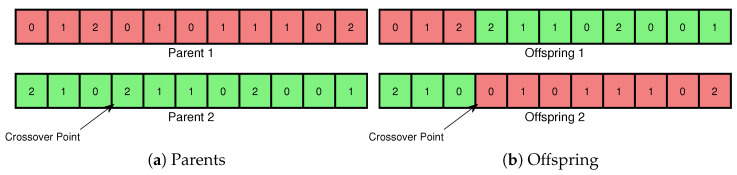
Virtual node-driven genetic algorithm crossover.

**Figure 5:**
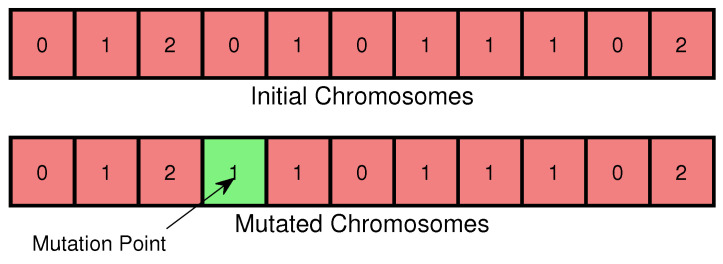
Virtual node-driven genetic algorithm mutation.

In the VND-GA algorithm, genes represent task assignment strategies, but there is no one-to-one correspondence between genes and strategies due to optimization during gene decoding. Chromosomes are encoded as integer arrays (0, 1, 2), where 0, 1, and 2 denote tasks assigned to the edge computing box, cloud server, and virtual node, respectively, following [27]. The fitness function employs the objective function of the virtual node-driven ILP algorithm.
**Algorithm 1** VND-GA**Initialize population**: Genes are randomly assigned values of 0 or 1 based on a random number threshold (0.5), and a correction process ensures constraints by modifying genes to 2 when edge or cloud resources are fully utilized.**Evaluate fitness**: Calculate the fitness score of each individual in the population.**repeat**   **Selection**: Select pairs of parents from the population based on their fitness.   **Crossover**: Perform single-point crossover to combine two chromosomes by exchanging genes around a selected point, generating two offspring (Figure 4).   **Mutation**: Apply mutation to the offspring. Each gene is mutated with equal probability to create potentially fitter individuals (Figure 5).   **Modification**: Traverse the offspring’s genes in random order; if a gene exceeds the edge or cloud resource limit, set it to 2.   **Replacement**: Replace the current population with the new offspring.**until** max generations meet **return** the best solution found.

### 5.3. VND-HA Algorithm

The advantage of heuristic algorithms lies in their ability to quickly find approximately optimal solutions without requiring extensive computation. When handling large-scale problems, heuristic algorithms often outperform traditional algorithms, such as genetic algorithms, particle swarm optimization, and simulated annealing, in terms of speed, and may even yield better results. The heuristic algorithm proposed by Lai et al. [32] for the EUA problem consists of two main components: first, tasks are sorted based on a custom priority; second, while ensuring the execution of all tasks, the algorithm increases resource allocation to tasks to achieve a higher total QoE, as described in Algorithm 2.

Inspired by the QoEUA algorithm, we propose a virtual node-driven heuristic algorithm (VND-HA) to solve the EUARS problem, as described in Algorithm 3. This algorithm also uses the priority of surveillance camera tasks for sorting. Additionally, it introduces virtual nodes to ensure that feasible solutions exist even under resource constraints.
**Algorithm 2** QoEUA [32]a set of edge servers *S*, a set of users *U*, and a set of QoS levels *W*all users $u_{j}$, $\forall u_{j}inU$, are unallocatedsort *U* in ascending order of the number of neighbor edge servers (i.e., users who are covered by fewer edge servers are prioritized, being the first to be allocated)**repeat**   **for** each user $u_{i}\in U$ **do**     $S\left(u_{i}\right)\overset{▵}{=}$ user $u_{i}$’s neighbor edge servers     allocate user $u_{i}$ to an edge server $s_{j}\in S\left(u_{i}\right)$ which has the most available capacity,     and increase user $u_{i}$’s current QoS level $W_{l}$ by one level, i.e., $W_{l+1}$   **end for****until** no users can improve their QoS levels

**Algorithm 3** VND-HA
all stream $u_{i}$, $u_{i}\in U$, are unallocatedsort U in descending order of priority**for** each stream $u_{i}\in U$ **do** **for** each edge server $s_{j}\in S$ **do**    **if** $s_{j}$ can connect with $u_{i}$$s_{j}$ has sufficient resources **then**       allocate $u_{i}$ to $s_{j}$       fresh resources of $s_{j}$    **end if** **end for** **if** c has sufficient resources **then**   allocate $u_{i}$ to *c*   fresh resources of *c* **else**   allocate $u_{i}$ to virtual node **end if**
**end for**



## 6. Experimental Evaluation

In this section, we evaluate performance of the proposed four algorithms through a series of experiments. All experiments were conducted on a Windows Desktop with an Intel Core i7-9750H processor and 16GB of RAM. The ILP algorithm outlined in Section 4 was solved using the Gurobi [33] 11.0.0 solver.

### 6.1. Experimental Settings

The video stream task set used for testing includes the following parameters for each task: $<u_{i},s_{j},p,D>$. The $u_{i}$ parameter uniquely identifies the *i*-th task. The $s_{j}$ parameter specifies the *j*-th edge server to which the monitoring camera is connected. Each monitoring camera is connected to one and only one edge server through a network cable. The *p* parameter represents the processing priority of the task, with priorities denoted by the numbers 1, 2, and 3, corresponding to low, medium, and high priority, respectively. The higher the priority of a video stream task, the more resources it will consume. Table 4 shows the resources required by tasks of different priorities.

### 6.2. Experimental Design

To evaluate the performance of the four proposed algorithms under both resource-abundant and resource-scarce scenarios, we conduct a total of 220 experiments, organized into 11 groups, with each group containing 20 experiments. The number of tasks increases across groups, while the computational resources of the edge computing boxes and the cloud server are kept constant throughout all experiments. For instance, test IDs 1 through 20 correspond to the same number of tasks, while test IDs 21 through 40 correspond to a larger number of tasks than those in 1 through 20. As the test ID increases, the total number of tasks grows monotonically, transitioning gradually from a resource-abundant scenario to a resource-constrained one.

Within each group of 20 experiments, the distribution of high-priority, medium-priority, and low-priority tasks is determined randomly. The distribution ratio of task priorities is approximately 1:3:6 for high, medium, and low priorities, with white noise added to account for variation. This experimental design allows for a comprehensive evaluation of the algorithms’ adaptability and robustness across different resource availability conditions.

**Figure 6:**
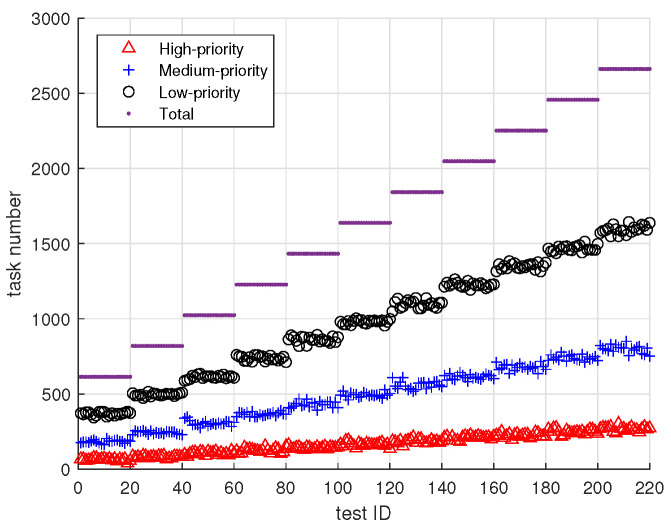
Test data graph. The x-axis represents the test ID, and the y-axis shows the task number. We conduct 220 experiments, divided into 11 groups of 20 experiments each. Each group has the same number of tasks, with task priorities distributed in a 1:3:6 ratio for high, medium, and low priorities.

Figure 6 illustrates the number of tasks in the experimental data set. In this data set, the total number of tasks ranges from 600 to 2600, encompassing both low-load and resource scarcity scenarios. There are 64 edge computing boxes, each equipped with 32 CPU cores, 4 GPUs, 64 Gb of memory, and 100 Mbps bandwidth. The private cloud has 512 CPU cores, 128 GPUs, 1024 Gb of memory, and 1000 Mbps bandwidth.

We will focus on the task scheduling success rate as the primary metric. The main goal is to prioritize the scheduling of higher priority tasks. A higher task scheduling success rate indicates better performance, as it signifies more tasks being successfully assigned to the edge servers. Additionally, we will assess the number of tasks assigned to the edge servers: the larger the number of tasks scheduled, the better the performance. Furthermore, we will examine the resource utilization of the edge servers, where higher resource utilization reflects more efficient use of the available resources. Finally, we will evaluate the computational efficiency of all algorithms. This comprehensive assessment will evaluate the four algorithms from the above four aspects. Note that the following VND-GA experimental results correspond to a population size of 50 and a genetic generation of 20. The population size (50) and the number of max generations (20) are determined by preliminary experiments. These values provide a balance between computational efficiency and quality of solution. Specifically, we observe that increasing the population size beyond 50 does not yield significant improvements in the quality of solution.

### 6.3. Experimental Results

By considering the following four aspects, i.e., task scheduling success rate, the number of tasks assigned to edge servers, edge server resource utilization, and computational efficiency, we can thoroughly evaluate the performance of the four algorithms and determine the most effective one. The random algorithm does not consider task priorities or the coordination between tasks and edge computing boxes. The VND-GA algorithm, due to not employing gradient descent, exhibits slower convergence. In the tested scenarios discussed in this paper, it is unacceptable for high-priority tasks to wait several minutes for sufficient computational resources to be allocated, leading to suboptimal results. The VND-HA algorithm adopts a locally greedy approach, processing tasks in order of priority without considering the coordination between tasks and edge computing boxes. Due to these limitations, all the random, VND-GA, and VND-HA algorithms perform worse than our proposed ILP algorithm.

#### 6.3.1. Task Scheduling Success Rate

**Figure 7:**
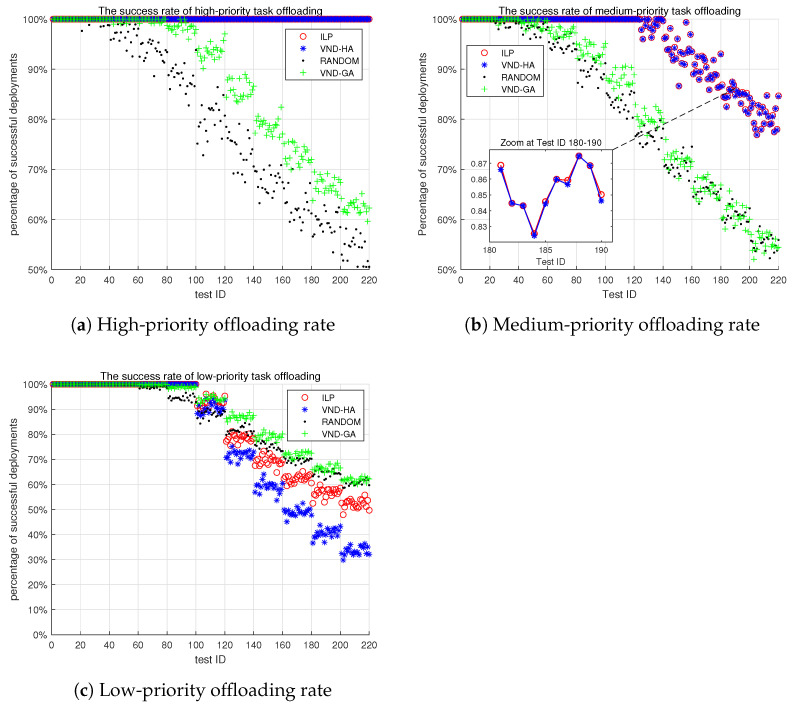
Task scheduling success rate. The x-axis represents the test ID, and the y-axis shows the percentage of successful task offloading.

Figure 7a illustrates the scheduling success rate for high-priority tasks of the four algorithms. Both the random and VND-GA algorithms start with a 100% success rate, but as the number of tasks increases, the success rate gradually decreases to around 50%. This decline occurs because, as shown in Figure 6, when the number of tasks exceeds 800, the initially abundant computational resources become severely constrained. In contrast, both the ILP and VND-HA algorithms maintain a 100% scheduling success rate for high-priority tasks. Even when computational resources are severely limited, these algorithms still prioritize the execution of high-priority tasks. This demonstrates the effectiveness of the ILP and VND-HA algorithms in ensuring the successful scheduling of critical tasks, even when the overall task load increases and resources become scarce. A key advantage of these two more sophisticated algorithms, compared to the random and VND-GA algorithms, is their ability to consistently prioritize high-priority tasks.

Figure 7b shows the scheduling success rate for medium-priority tasks of the four algorithms. Until the 120th experiment, both the ILP and VND-HA algorithms achieve a 100% success rate for medium-priority task scheduling. In contrast, the random and VND-GA algorithms exhibit a similar declining trend as seen for high-priority tasks. However, after the 120th experiment, when the total number of tasks exceeds 1800, the inherent lack of computational resources prevents both the ILP and heuristic algorithms from fully ensuring 100% scheduling success for medium-priority tasks.

Notably, in scenarios with severe resource constraints, the ILP algorithm slightly outperforms the VND-HA algorithm in the scheduling success rate of medium-priority tasks. This underscores the exceptional performance of the proposed ILP algorithm. The results highlight the ability of both the ILP and VND-HA algorithms to prioritize medium-priority tasks and maintain a high success rate of scheduling, even when the overall task load increases and resources become scarce.

Figure 7c presents the scheduling success rate for low-priority tasks of the four algorithms. This further emphasizes the superiority of the ILP algorithm over the VND-HA algorithm. As computational resources become increasingly limited, it is evident that the ILP algorithm significantly outperforms the VND-HA algorithm in terms of low-priority task scheduling success.

#### 6.3.2. Number of Tasks Assigned to Edge Servers

Scheduling tasks to the edge servers, as opposed to a centralized cloud, results in decreased transmission delays and quicker response times. This proximity reduces network latency involved in data transmission, thereby improving overall user experience and system responsiveness.

**Figure 8:**
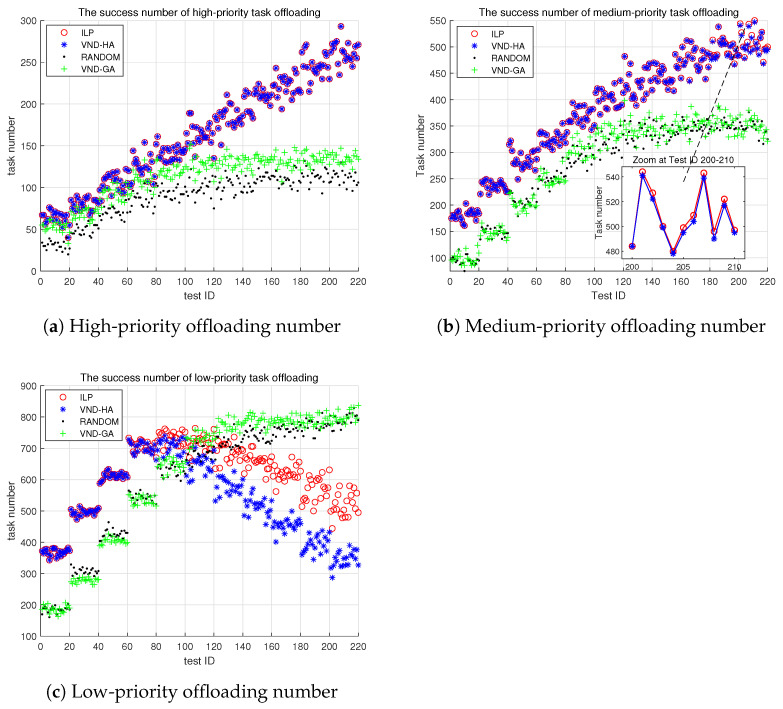
The number of tasks offloaded to edge servers. The x-axis represents the test ID, and the y-axis shows the number of tasks offloaded to servers.

In Figure 8a, we observe that the number of high-priority tasks scheduled on the edge servers is very similar between the ILP and VND-HA algorithms. In terms of edge server scheduling for high-priority tasks, they are essentially comparable. However, in Figure 8b, a notable change occurs once the test ID exceeds 100. At this point, the ILP algorithm schedules significantly more medium-priority tasks to the edge servers compared to the VND-HA algorithm, and as the test ID increases further, this difference becomes increasingly pronounced. Importantly, in terms of scheduling high-priority and medium-priority tasks to the edge server, both the ILP and VND-HA algorithms outperform the random and VND-GA algorithms.

The results in Figure 8 indicate that the ILP algorithm has an obvious advantage over the VND-HA algorithm when it comes to optimizing edge server scheduling for medium-priority and low-priority tasks, particularly as the overall task load increases. Furthermore, the ILP algorithm outperforms both the random and VND-GA algorithms in optimizing the edge server scheduling of high-priority and medium-priority tasks.

#### 6.3.3. Edge Server Resource Utilization

The resource utilization rate on the edge side is a crucial indicator of the performance of resource scheduling algorithms. A higher resource utilization rate indicates better performance of the scheduling algorithm.

**Figure 9:**
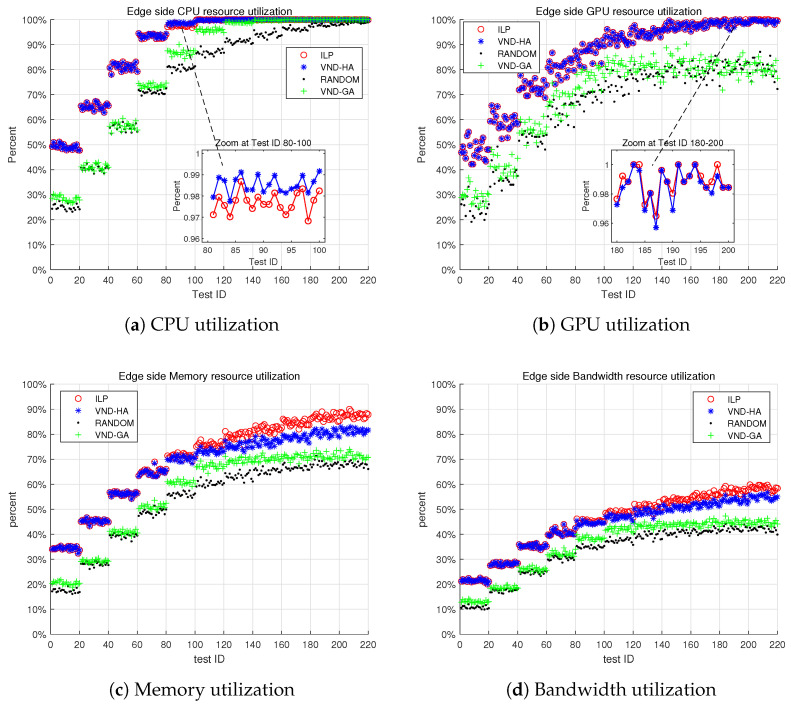
Edge server resource utilization. The x-axis represents the test ID, and the y-axis shows the resource utilization.

Figure 9a shows the CPU utilization on the edge server. It is evident that when the test ID exceeds 100, the edge CPU resources become congested. The ILP, VND-HA, and VND-GA algorithms all achieve near 100% CPU utilization, while the random algorithm reaches close to 100% utilization only after the test ID surpasses 200.

Figure 9b illustrates the GPU utilization on the edge server. Compared to the CPU, the GPU data vary more, with GPU resource shortages only occurring when the test ID exceeds 140. The reason for this difference is that in the test scenario, we assume that the edge server may only have a single CPU, and most tasks on the edge server are CPU-intensive. As a result, fewer tasks require GPU resources.

Figure 9c,d present the memory and network bandwidth utilization on the edge, respectively. Compared to the other three algorithms, the ILP algorithm significantly improves resource utilization.

#### 6.3.4. Computational Efficiency

**Figure 10:**
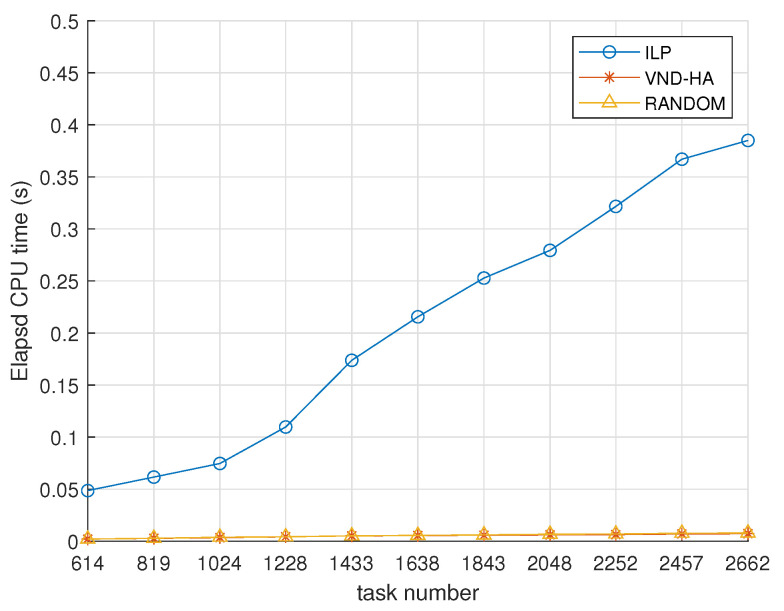
CPU execution time. The x-axis represents the total number of tasks, and the y-axis shows the execution time of the algorithms in seconds.

Figure 10 demonstrates the efficiency of the ILP, VND-HA, and random algorithms, measured by CPU execution time. The VND-GA algorithm is not included in the comparison because it is an iterative method, and its execution time depends on the number of iterations. As shown in the experimental results presented earlier, the time consumed by the VND-GA algorithm significantly exceeds that of the ILP algorithm.

As mentioned earlier, we conduct a total of 220 experiments, organized into 11 groups, with each group containing 20 experiments and the same total number of tasks. To compare the efficiency of the above three algorithms, we perform 100 repeated runs for each experiment within a group and calculate the average runtime of a group. Thereafter, we further calculate the mean of these averages across all 20 experiments in the group, yielding the overall runtime for a given number of tasks. It can be seen from Figure 10 that, as the total number of tasks increases, the elapsed CPU time of all three algorithms also increases. Furthermore, among the three compared algorithms, the ILP algorithm takes the longest time. In the experimental group with 2662 tasks, the ILP algorithm requires an average of 0.38 s to complete the resource allocation.

In real scenarios, high-priority tasks often involve video surveillance stream data that require special attention and have a certain degree of inertia. Therefore, a computing delay of 0.38 s is acceptable and meets the need for real-time allocation of computing resources.

## 7. Conclusions

In this study, we address the EUARS problem and make several significant contributions. First, we formally define the concept of the virtual node in addressing the EUARS problem. This concept renders a universal architecture for addressing resource scarcity, compared to the existing work in Table 1. The new virtual node formulation allows a more robust and efficient allocation of tasks by introducing abstract physical resources into manageable units. Then we propose an optimal scheduling algorithm by using integer linear programming (ILP) to solve the EUARS problem exactly. By prioritizing tasks based on their importance, the ILP algorithm ensures that high-priority tasks receive the necessary resources promptly, enhancing the overall efficiency and responsiveness of the system.

Through numerical simulation experiments, we have compared the performance of the ILP algorithm with three other comparative algorithms, i.e., the VND-HA, VND-GA, and random algorithms. We show that the ILP algorithm significantly outperforms the other three algorithms in terms of task scheduling success rate, as shown in Figure 7, and edge resource utilization, as shown in Figure 8. Specifically, the ILP algorithm demonstrates superior performance in utilizing CPU, GPU, memory, and network bandwidth resources on edge computing boxes, as shown in Figure 9.

While the ILP algorithm achieves near-optimal resource allocation, it does incur higher computational costs, as evidenced by the elapsed CPU time. Despite this, the delay is still within acceptable limits for real-time applications, particularly for high-priority tasks that require immediate attention.

In summary, the ILP algorithm not only optimizes resource utilization but also ensures that critical tasks are prioritized effectively. Our study highlights the potential of ILP in solving complex resource allocation problems in edge computing environments, providing a robust framework for future research and practical implementations in real-world scenarios.

We acknowledge that our study still has some limitations. First, our experiments are conducted in a simulated environment, which may not fully capture the complexities of real-world edge computing systems. For example, we omit the virtual machine setup time on the edge computing box and reusable scheduling for identical tasks. Second, the proposed ILP algorithm assumes that task priorities are known in advance, which may not always be the case in practical scenarios.

In future work, we plan to validate our ILP algorithm in real-world edge computing offloading to address the limitations of simulation-based evaluation. Additionally, we will explore the application of virtual nodes in MEC, smart city, and smart building scenarios to optimize the topology of the resource scheduling model and enhance the robustness of the algorithm. For example, in the field of autonomous driving, safety detection can be assigned the highest priority, while tasks such as path planning are given lower priority. By introducing virtual nodes, robust scheduling of tasks with different priorities can be achieved.

## Figures and Tables

**Table 1:** Comparison of related work in architecture, resource scarcity, and task priority.

Work	Architecture	Resource Scarcity Considered	Task Priority Considered
Ning et al. [18]	user–edge	No	No
Chen et al. [19]	user–edge	No	No
Lyu et al. [20]	user–edge	No	Yes
Yang et al. [21]	user–edge–cloud	No	No
Li et al. [22]	user–edge	No	No
Deng et al. [23]	user–edge	Yes	No
Xu et al. [24]	user–edge–cloud	No	No
Qiao et al. [25]	user–edge	No	No
Xing et al. [26]	user–edge	No	No
Xu et al. [27]	user–edge–cloud	No	No
Yu et al. [28]	user–edge–cloud	No	No
Huang et al. [29]	user–edge–cloud	No	No
Badri et al. [30]	user–edge	No	No
Li et al. [31]	user–edge	Yes	Yes
Lai et al. [32]	user–edge	No	No
VND-ILP	user–edge–cloud	Yes	Yes
VND-GA	user–edge–cloud	Yes	Yes
VND-HA	user–edge–cloud	Yes	Yes

**Table 2:** List of notations.

Symbol	Description
*m*	number of edge servers
*n*	number of monitor surveillance tasks
*C*	cloud server
*S*	edge computing boxes
$s_{i}$	the *i*-th edge computing box
*U*	surveillance cameras
$u_{i}$	the *i*-th surveillance camera task
*D*	a set of computing resource types ^1^
$D_{c,k}$	the *k*-th ^2^ type of computing resources in the cloud
$D_{s_{i},k}$	the *k*-th ^2^ type of computing resources in the *i*-th edge computing box
$D_{u_{i},k}$	the *k*-th ^2^ type of computing resources required in the *i*-th surveillance camera task
$p_{i}$	the *i*-th ^3^ priority
$x_{i,j}$	myredboolean variable indicating whether the *i*-th surveillance camera task is scheduled on the *k*-th edge computing box
$y_{i}$	myredboolean variable indicating whether the *i*-th surveillance camera task is scheduled on the cloud server
$v_{i}$	myredboolean variable indicating whether the *i*-th surveillance camera task is scheduled on the virtual node
*g*	a hyperparameter distinguishing task rewards by priority
*k*	a hyperparameter capturing edge–cloud latency differences

^1^ Computing resources in this study mainly include CPU cores, GPU blocks, memory, and bandwidth. ^2^ k=1 denotes CPU cores, k=2 denotes GPU blocks, k=3 denotes memory, and k=4 denotes bandwidth. ^3^ i=1 denotes a low-priority task, i=2 denotes a middle-priority task, and i=3 denotes a high-priority task.

**Table 3:** Typical solutions to the problem in Figure 3.

Camera 1			Camera 2			Camera 3			Camera 4			Camera 5			Camera 6			Reward
$x_{1,1}$	$y_{1}$	$v_{1}$	$x_{2,1}$	$y_{2}$	$v_{2}$	$x_{3,1}$	$y_{3}$	$v_{3}$	$x_{4,1}$	$y_{4}$	$v_{4}$	$x_{5,1}$	$y_{5}$	$v_{5}$	$x_{6,1}$	$y_{6}$	$v_{6}$	
1	0	0	1	0	0	0	1	0	0	1	0	0	1	0	0	0	1	9.5
0	1	0	1	0	0	1	0	0	0	1	0	0	1	0	0	0	1	5
0	0	1	1	0	0	1	0	0	0	1	0	0	1	0	0	1	0	−2.5

**Table 4:** Task priority and resource requirement comparison table.

Priority	CPU	GPU	Mem (Gb)	Bandwidth (Mbps)
1	1 or 2	0	1 or 2	0.5 or 2
2	1 or 2	0 or 1	2 or 4	2 or 4
3	2 or 4	1	4 or 8	4 or 8

## Data Availability

Data are contained within the article.

## Abbreviations

The following abbreviations are used in this manuscript:

QoS	Quality of Service
ILP	integer linear programming
IoT	Internet of Things
QoE	Quality of Experience
EUARS	Edge User Allocation under Resource Scarcity
DPCOEM	Dynamic Parallel Computing Offloading and Energy Management
OREO	Online seRvice caching for mobile Edge cOmputing
MINLP	Mixed-Integer Nonlinear Programming
NSGA-III	Non-Dominated Sorting Genetic Algorithm III
D2D	device-to-device
MILP	mixed integer linear programming
SCA	Successive Convex Approximation
UAVs	unmanned aerial vehicles
SAA	Sample Average Approximation
EUA	Edge User Allocation
GA	genetic algorithm
VND-ILP	virtual node-driven integer linear programming
VND-GA	virtual node-driven genetic algorithm
VND-HA	virtual node-driven heuristic algorithm
MEC	mobile edge computing
